# A Simple and Compact MR-Compatible Electromagnetic Vibrotactile Stimulator

**DOI:** 10.3389/fnins.2019.01403

**Published:** 2020-01-17

**Authors:** Xinjian Jiang, Yueqian Wang, Xiaojin Li, Liping Wang, Yong-Di Zhou, Huimin Wang

**Affiliations:** ^1^Key Laboratory of Brain Functional Genomics (MOE & STCSM), School of Psychology and Cognitive Science, Institute of Cognitive Neuroscience, East China Normal University, Shanghai, China; ^2^Department of Electronic Engineering, School of Information Science and Technology, East China Normal University, Shanghai, China; ^3^Key Laboratory of Primate Neurobiology, CAS Center for Excellence in Brain Science and Intelligence Technology, Institute of Neuroscience, Chinese Academy of Sciences, Shanghai, China

**Keywords:** electromagnetic vibrator, functional magnetic resonance imaging, frequency discrimination task, vibrotactile, MR-compatible

## Abstract

We have developed a low-cost electromagnetic vibrotactile stimulator that uses the magnetic field of an MR scanner as a permanent magnet to power a vibrating motor. A simple variable current power supply is controlled by software using a USB data acquisition controller. In our study, the function of our novel stimulator was verified in a vibration frequency discrimination working memory task, in which various ranges of frequencies and amplitudes are delivered in MRI scanner. Furthermore, our functional MRI study revealed activations of the primary and secondary somatosensory cortices during the perception of tactile stimulation. Therefore, the new designed electromagnetic vibrotactile stimulator is capable of generating various frequencies of tactile stimuli and represents a powerful and useful tool for studying somatosensory functions with functional MRI.

## Introduction

Tactile senses play a very important role in daily life, such as the ability to discriminate between different materials of furniture or clothing (Kaas et al., 2013; Kim et al., 2019). Tactile-related cognitive functions, such as tactile memory, also play a crucial role in people’s lives, including the ability to remember the sharpness of a knife (Gallace and Spence, 2009; Katus and Eimer, 2019). Furthermore, tactile information can be used to identify and operate objects, particularly in situations such as firefighting or braille reading, where one cannot access information visually (Heller, 1989; Grant et al., 2000). Therefore, both tactile sensation and tactile memory are important cognitive functions that lay the foundation for the integration of processing different types of sensory information.

Vibrational stimuli with different frequencies have traditionally been used in tactile research (Romo and Salinas, 2003; Barak et al., 2010). As early as the 1960s, Mountcastle et al. (1967) investigated the tactile and perceptual encoding of vibrational stimuli with different frequencies using single-cell electrophysiological recordings (Talbot et al., 1968). After comparing the results of psychophysical experiment between humans and macaques, they found that humans and macaques exhibit similar abilities to perceive and distinguish tactile vibrational frequencies (LaMotte and Mountcastle, 1975). Subsequently, Romo et al. studied the neurophysiological mechanisms of tactile working memory in different brain regions of primates using vibrotactile stimuli (Romo et al., 1999; Luna et al., 2005). Our team has been investigating haptic-related high-level cognitive function in recent years, and we have found that various brain regions are involved in haptic working memory and decision making (Zhou and Fuster, 1996, 2000). Studies using transcranial magnetic stimulation identified a key role for contralateral primary somatosensory cortices in a vibrotactile working memory task (Harris et al., 2002; Zhao et al., 2017). Electroencephalography studies have found that vibration induces unique neuronal firing patterns in the primary somatosensory cortex in response to different vibrotactile frequencies (Giabbiconi et al., 2007; Spitzer et al., 2010). These studies have substantially enhanced our understanding of the neural mechanisms underlying tactile sensory perception and working memory. With the development of brain imaging techniques, an increasing number of researchers have sought to investigate the neural mechanisms of vibrotactile perception, working memory, and decision making (Pleger and Villringer, 2013; Malone et al., 2019). In the past few years, some functional magnetic resonance imaging (fMRI) experiments have also explored the neural mechanisms of vibrotactile frequency discrimination (Preuschhof et al., 2006; Hegner et al., 2010; Seri et al., 2019). Owing to the high spatial resolution of fMRI, this technique is particularly useful for investigations of tactile sensation and its relevant cognitive functions at the whole-brain level. Changes in the frequency, intensity, duration, and location of tactile stimuli can cause various activation patterns in different brain regions (Jones and Sarter, 2008). However, the development of an apparatus that presents tactile stimuli has been restricted by the high complexity and prices of tactile mechanical stimulators compared with the presentation of other stimulus modalities (visual or auditory stimuli). Tactile stimulators are complex because they must be able to operate with stability in a magnetic resonance (MR) environment without affecting MR images. Therefore, an issue of great scientific significance is the development of an MR-compatible vibrotactile stimulator that can be quantitatively controlled.

Vibrotactile stimulators have been widely applied in the MR environment (Hartwig et al., 2017). The types of vibrotactile stimulators used for fMRI studies include electromagnetic (Graham et al., 2001; Kim et al., 2013), pneumatic (Chakravarty et al., 2009; Gallasch et al., 2010; Goossens et al., 2016), and piezoelectric (Harrington et al., 2000; Gizewski et al., 2005; Tamè and Holmes, 2016; Seri et al., 2019) stimulators, in addition to other types. The electromagnetic type of stimulator can generate a vibrating stimulus using the magnetic field of the MR scanner. The electromagnetic tactile stimulation device is designed using winding coils (Graham et al., 2001), but the actuator is large and not suitable for applying multichannel stimuli. Another stimulator device, which was designed using a planar coil actuator (Kim et al., 2013), has a wide range of stimulation frequencies and stimulation intensities; however, the drive unit located inside the MR scanner room may be affected by the magnetic field. The pneumatic type of stimulator has been used to apply normal indentations by injecting air on the skin (Wienbruch et al., 2006) and has also been used to apply vibrotactile stimulation by controlling the air flow (Chakravarty et al., 2009; Montant et al., 2009). These stimulators have low spatial resolution, and the stimulus intensity is not well controlled. Moreover, the design is often complex and requires the concomitant use of several types of materials and different electronic subsystems. The piezoelectric type stimulator is equipped with a large contact area, and thus, the stimulation of a precise location, such as one finger, is difficult (Harrington et al., 2000). In addition, the structure of the piezoelectric hardware is complicated.

In the current study, we developed a low-cost (approximately $500), simple, and compact electromagnetic vibrotactile stimulator. It can be used to apply a vibrating stimulus using the magnetic field of the MR scanner. The coil current is variably adjusted by the data acquisition device to change frequency and amplitude of vibration. When the stimulator is charged with electric current, the maximum vibration frequency can reach 100 Hz. The vibration amplitude is adjustable and can be changed without affecting the frequency. The stimulator consists of a data acquisition device, an amplification circuit, and an actuator. The actuator is composed of copper wire and plastic. Shielded copper cables connect to both the amplification circuit, which is located outside the scanner room, and actuator, which is located in the scanner room. These materials (copper and plastic) show very low susceptibility to magnetic fields and thus can be safely used in the MR environment since the interactions with both the static and dynamic components of the magnetic field are negligible (Hartwig et al., 2017). This study provides a detailed introduction and guide for the application of our newly developed vibrotactile stimulator. The results of behavioral and fMRI experiments support the effectiveness of our stimulator in an experimental setting.

## Materials and Methods

### Theory

Vibrotactile stimulator is, in essence, similar to a magnetoelectric ammeter. Magnetoelectric ammeters are manufactured based on the principle that current-carrying rectangular coils rotate with the electromagnetic force in a magnetic field. The coil drives the meter needle to deflect when the desired current passes through the ammeter coil. The vibration stimulator we have invented uses the magnetic field of the MR scanner as the permanent magnet. A rectangular coil that goes back and forth around the axis is installed on the stimulator. The electromagnetic force applied to the coil is proportional to the strength of current and the size of coil.

The principle behind the force coil is the Lorentz force law [Equation (1)], which states that the force on a current-carrying wire perpendicular to a static magnetic field is proportional to the length *l* of the wire, the size *B* of the field, and the magnitude *I* of the current. A coil with *n* turns of wire will produce *n* times the amount of current when pivoted at one end.

(1) F=nBIl

For our stimulator, *n* = 23, *B* = 3 T, *I* = 2 A, *l* = 1.485 cm, and *F* = 23 × 3 × 2 × 1.485 × 10^–2^ = 2.050 N.

### Hardware

**FIGURE 1:**
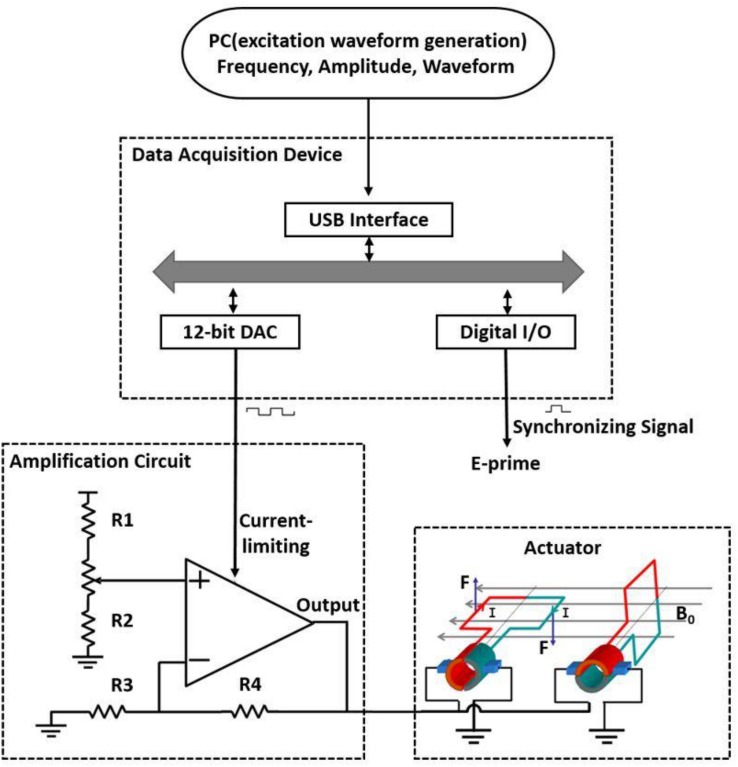
Schematic of a simple magnetic-resonance-compatible vibrotactile stimulator. The PC generates and transfers the waveform data to be converted; the data are then parameterized according to frequency, amplitude, and duration and sent to the data acquisition device through the USB interface. The data acquisition device converts the received data into an analog signal through a 12-bit digital-to-analog converter (DAC) and exports the signal to the current limiter of the amplification circuit. At this time, the E-prime software receives a synchronization pulse. The output of the amplifier changes with the degree of current limitation, thus generating various ampere forces on the coil to drive the rotation of the coil. The actuator is located inside the MR scanner room, and other devices are located outside the scanner room. R1–R4, resistors, R1 = 5 kΩ, R2 = R3 = R4 = 10 kΩ; *B*_0_, magnetic field; *I*, current; *F*, Ampère’s force.

The hardware system of this vibrotactile stimulator is shown in Figure 1. It is mainly comprised of a computer control terminal, a DT9812 data acquisition instrument (Measurement Computing Corporation, United States), an amplifier circuit (OPA 548T), and a vibrator coil. The computer generates and transfers the waveform data to the data acquisition instrument through the USB interface to be converted and parameterized according to the frequency, amplitude, and duration of stimulation. The data acquisition instrument converts received data into an analog signal through a 12-bit digital-to-analog converter (DAC) and exports the signal to the current limiter of the amplification circuit. At this point, the software E-prime receives a synchronization pulse. The current output of the amplifier changes with the degree of limiting current, thus generating a varying ampere force on the coil to drive the rotation of the coil.

**FIGURE 2:**
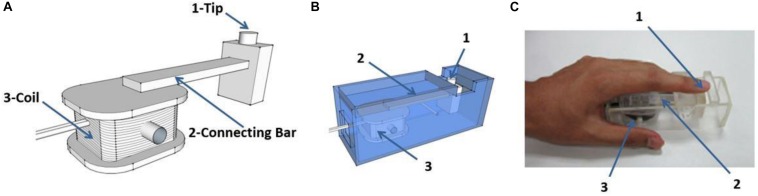
Schematic diagram of the stimulator. **(A)** The rectangular coil connects a bar which is positioned perpendicular to the coil axis. The coil rotates under the effect of ampere-force and drives the connecting bar to move together, and contacts the finger through the tip of the stimulator. **(B)** The coil is placed in a plastic box (material, polymethyl methacrylate). **(C)** Example of positioning of a participants’ finger during experiments. The vibration stimuli are applied on the pad of index finger. “1,” “2,” and “3” in **(B,C)** as indicated in **(A)**.

The dimensions of the rectangular coil are 30 mm × 20 mm × 10 mm and comprise 40 coiled wires. The rectangular coil connects a perpendicular 70 mm long bar to the coil axis. The diameter of the stimulator tip, which contacts a participant’s finger, is 5 mm (Figure 2A). The coil is placed in a plastic box with dimensions of 110 mm × 40 mm × 35 mm (Figure 2B). The coil rotates as a result of Ampère’s force in the magnetic field when current passes through the rectangular coil, which drives the connecting bars to move together, thus producing a vibrational stimulus. A gap of 15 mm is left at the top of the organic glass box so that the participant’s fingers can easily contact the tip of the stimulator (Figure 2C).

### Software

The C++-based software is designed to deliver stimuli in a block protocol. A simple graphical user interface controls the setup and timing of stimulus presentation. The rest time, stimulation time, and number of blocks are set by the graphical user interface. The frequency and intensity of stimulation are also controlled by the software. The frequency can reach 100 Hz. The output of the stimulation signal is a square wave.

**FIGURE 3:**
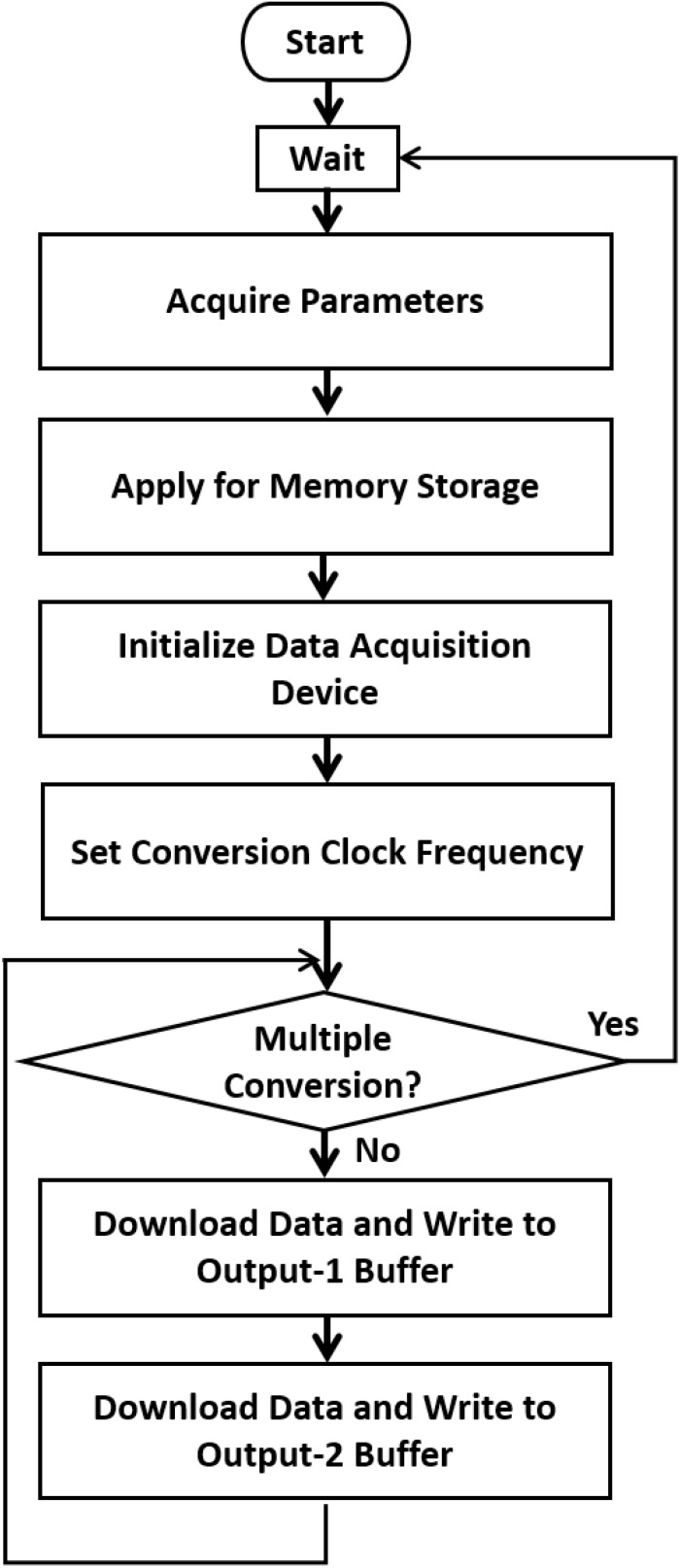
A single vibration scenario. When a test begins, the system (1) acquires the parameters related to the stimulus, such as the total repeated period, vibration frequency, duty cycle, and amplitude. (2) Creates memory storage and generates the data to be converted according to the desired conversion frequency. (3) Initializes the data acquisition device and sets the conversion clock frequency. (4) Downloads the data through the USB port to the device, repeatedly writes to the two output buffers, starts the conversion, and waits until the conversion is finished. When multiple conversions are required, the Microsoft Foundation Classes controlling program can be modified.

The system consists of two components: the parameter setting and controlling component, which is based on Microsoft Foundation Classes, and the component that controls the function of the data acquisition device. Figure 3 depicts a single vibration scenario. The data and software code that support the findings of this study are available from the corresponding author upon reasonable request.

### Stimulation Parameters

The output of the stimulation signal is a square wave. The stimulation duration and the interval between the stimuli can be precisely controlled to 0.1 s. The stimulation frequency can reach 100 Hz. The stimulation intensity is controlled by the current magnitude, which ranges from 0 to 5 A.

### fMRI Experiments

A pilot fMRI experiment was conducted to test the applicability of the developed stimulator.

#### Experiment 1: Vibrotactile Frequency Perception

Six right-handed healthy college students [2 female, 24.8 ± 1.3 (mean ± SD) years old] participated in this experiment. The study protocol was approved by the Committee on Human Research Protection at East China Normal University, and informed consent was obtained individually from all participants.

**FIGURE 4:**
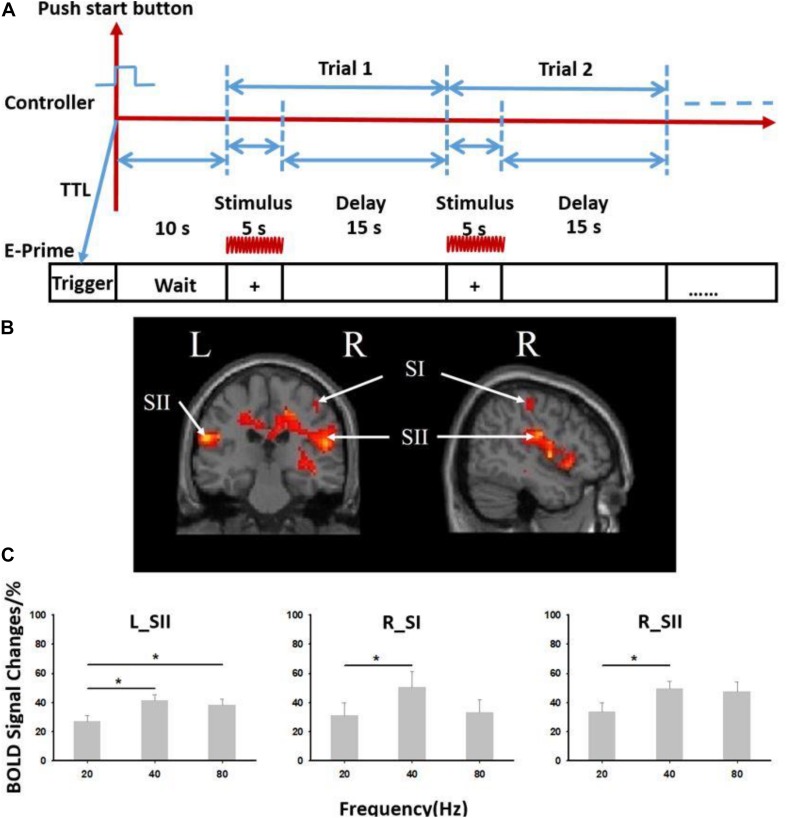
Results from six subjects used in the vibrotactile frequency perception task. **(A)** Paradigm used for experiment. The 5-s ON (stimulation phase)–15-s OFF (resting phase) cycle was adopted for stimulus presentation. **(B)** Brain activation from six subjects used in the experiment. **(C)** Blood oxygenation level-dependent (BOLD) signal changes at different stimulation frequencies (20, 40, and 80 Hz). R, right; L, left; SI, primary somatosensory cortex; SII, secondary somatosensory cortex. ^∗^Paired *t* test, *p* < 0.05. Error bars indicate standard deviation from the mean.

The experiment consisted of four blocks of vibrotactile stimulation tests. Each block involved both stimulation and resting phases. In the stimulation phase, three stimuli of different frequencies (20, 40, and 80 Hz) were randomly applied to the pad of the right index finger, and no stimulus was applied in the resting phase. All subjects were asked to close their eyes and wear a headset to prevent any disturbances from the surrounding environment. In each block, a 5-s ON (stimulation phase)–15-s OFF (resting phase) cycle was adopted for stimulus presentation (Figure 4A). Each block included 16 cycles.

The stimulus frequency, duration, and interval were controlled by the self-programing software. The alignment of the time of stimulus presentation and the time the MR scanner began acquiring data was determined by the E-prime software (Psychology Software Tools Inc., Sharpsburg, PA, United States). First, a trigger signal was sent to E-prime when the MR scanner began to scan. Then, a ready interface occurred in E-prime software. The experimenter began to control the stimulator using the self-programing software mentioned above. A transistor–transistor logic signal was then simultaneously sent to the E-prime software. This procedure eventually aligned the time of the stimulator, MR scanner, and E-prime software.

Neuroimaging data were acquired using a 3-T MRI system (Siemens Medical Systems, Erlangen, Germany) equipped with a standard 32-channel head coil. A high-resolution (1 mm × 1 mm × 1 mm voxel size) T1-weighted structural MRI was first acquired using a 3-D FLASH sequence for spatial registration and anatomical overlay of the functional data (TR = 1,900 ms, TE = 3.42 ms, matrix = 256 × 256, field of view = 240 mm × 240 mm, flip angle = 9°, slice thickness = 1 mm). Functional images were then obtained using a T2^∗^ weight single-shot gradient-recalled echo planar imaging (EPI) sequence, with 32-axial slices parallel to the AC–PC plane and coving the whole brain (TR = 2,000 ms, TE = 30 ms, matrix = 64 × 64, field of view = 240 mm × 240 mm, flip angle = 70°, slice thickness = 5 mm, in place resolution 3.75 × 3.75 mm). The three initial volumes in each run were discarded to avoid T1 saturation effects.

Standard preprocessing of the fMRI data was performed using SPM8 (Statistical Parametric Mapping, Wellcome Department of Imaging Neuroscience, UCL, London, United Kingdom) (Friston et al., 1994, 1995). Data preprocessing included corrections for the interlayer time and head motion, structural and functional image alignment, spatial standardization, spatial smoothing, and parameter estimation). Functional EPI data were first time shifted to the middle slice within a volume to correct for slice timing difference; time series in each run were then motion corrected using a local Pearson’s correlation. The EPIs were also aligned with individual anatomical data and warped to standard Talairach space using the TT_N27 template. Date were blurred within EPI mask with 4 mm full width at half maximum kernel to increase signal/noise ratio, scaled by the mean intensity of each run then multiply 100, and resampled into 3 × 3 × 3 mm voxels. Three events, including stimulus 1 (20 Hz), stimulus 2 (40 Hz), and stimulus 3 (80 Hz), were deconvoluted using the generalized linear model. The corresponding signals were excluded for time points in which the translation was >2 mm, and the rotation angle was >2°. Finally, we calculated the regression parameters and the significance level (*T*/*P* values) of each voxel for the three events. A one-sample *t* test was performed on the functional imaging data for all subjects to detect the activation at the group level, thus obtaining the average response and corresponding significance level of each voxel across three events. We then defined region of interest at contralateral primary somatosensory cortices (SI) and bilateral secondary somatosensory cortices (SII). ROIs were draw as 5-mm radius spheres whose center was the maximum event response voxel; the total volume of each ROI was 523.60 mm^3^.

#### Experiment 2: Vibrotactile Frequency Discrimination

Six right-handed healthy college students [4 female, 23.8 ± 1.6 (mean ± SD) years old] participated in this experiment. The study protocol was approved by the Committee on Human Research Protection at East China Normal University, and informed consent was obtained individually from all participants.

We designed a vibrotactile frequency discrimination task to verify whether the participants were able to distinguish different vibration frequencies produced by the tactile stimulator from one another. Each trial began with a 1-s presentation of an auditory stimulus. Subjects got the auditory stimulus, which was produced by E-prime through headphone. The auditory stimulus is pure tone of 1,600 Hz. A tactile vibration [stimulus 1 (S-1), 2.5-s duration] was applied to the participant’s left index finger. The frequencies of the vibration were 40, 60, 80, and 100 Hz. The four vibration frequencies differ from each other largely. For each trial, the set reference and comparison stimuli were chosen randomly from each of four frequencies. A delay of 5 s was implemented after the presentation of S-1. During the delay, participants were asked to think about the frequency of S-1. Then, the second tactile vibration [stimulus 2 (S-2), 2.5-s duration] was also applied to the left index finger. The two stimuli chosen in one trial were always different. The two-alternative forced choice (2-AFC) task was designed to test discrimination of vibration frequency. Participants were asked to report which vibration frequency was higher by pressing one of the two buttons corresponding to the placement of either their right index finger or their right middle finger as accurately and quickly as possible. The button assignment (e.g., the right index finger when S-1 is higher or the right middle finger when S-2 is higher) in experiments was counterbalanced across participants. A complete trial ended with the response. The intertrial interval was 4 s. Participants performed three experimental blocks, each of which contained 24 trials that were pseudorandomly assigned to vibrations with four different frequencies.

## Results

### Results of the Passive Stimulation Task

The fMRI data revealed significant activation in somatosensory cortices (both contralateral SI—“R_SI” and bilateral SII—“R_SII,” “L_SII,” false discovery rate *p* < 0.05 corrected, cluster size >20) (Figure 4B and Supplementary Table S1). A significant difference in the blood oxygenation level-dependent signal induced by stimuli of three different frequencies (20, 40, and 80 Hz) was also observed (Figure 4C). When the stimulation frequency was low (20 or 40 Hz), the activation increased as the frequency increased. When the stimulus frequency increased to 80 Hz, the activation decreased but was still significantly higher than that induced by the 20 Hz stimulus (*p* < 0.05).

### Results of the Discrimination Task

Six subjects participated in a vibrotactile frequency discrimination task and scored an average accuracy rating of 84.5 ± 5.2% (mean ± SD), indicating that the vibration stimuli were both effective and discernible (Supplementary Table S2 and Supplementary Figure S1).

## Discussion

The most significant contribution of this study is that we developed a tactile stimulation system using relatively simple devices. This system generates a series of tactile stimuli with various vibration frequencies in the MR environment. Using fMRI, although the number of subjects tested is small, we observed significant changes in brain activation upon the presentation of stimuli of different frequencies. Furthermore, the cost of system hardware, including electronic components and USB controllers, is far less than that of commercial stimulators. These electronic components are controlled by software, thus making the system efficient and user-friendly.

The tactile stimulator that we designed has many advantages compared with other pneumatic (Chakravarty et al., 2009; Gallasch et al., 2010; Goossens et al., 2016) and piezoelectric (Harrington et al., 2000; Gizewski et al., 2005; Tamè and Holmes, 2016) vibrators applied under the MR conditions. The stimulator is composed of a computer control terminal, a DT9812 data acquisition instrument, an amplification circuit (OPA548T), and a vibrator coil. Our system is small, simple, and inexpensive and has a wide variety of applications. In particular, since the stimulator is controlled with E-Prime software, tactile, auditory, and visual stimuli can be generated simultaneously.

The blood oxygenation level-dependent signals elicited by the stimuli varied across the three different frequencies presented (20, 40, and 80 Hz). Low (flutter, 5–40 Hz)- and high (vibration, 60–300 Hz)-frequency vibrating stimuli are perceived by two different receptors (Talbot et al., 1968). The Pacinian corpuscle is sensitive to high-frequency vibrations, and Meissner’s corpuscle, which is most concentrated in thick hairless skin, particularly the finger pads, is sensitive to low-frequency vibration (Talbot et al., 1968; Kandel et al., 2000). In the present study, the vibrational stimuli applied at frequencies of 20 and 40 Hz were considered low-frequency stimuli, and 80 Hz was considered a high-frequency stimulus. Therefore, when the stimulation frequency is low (20 or 40 Hz), the activation increased as the frequency increased. When the stimulus frequency increased to 80 Hz, the activation decreased. In an MRI study of periodontal ligament mechanoreceptors published in 2010, experimenters applied vibration stimuli to participant’s teeth at frequencies of 20, 50, and 100 Hz. Consistent with our results, the authors observed significant activation of the primary somatosensory and secondary somatosensory cortices, supplementary motor area, and posterior and anterior insular and parietal regions at low frequency (20 Hz). The activation of the primary and secondary somatosensory cortices was significantly reduced at higher frequencies (50 and 100 Hz) (Trulsson et al., 2010). These findings support the data presented herein.

Although the stimulation frequency is tightly controlled, the intensity of the stimulation changes with the orientation in the scanner. If the placement of the vibrating motors in the magnetic field changes, the intensity of the vibration also changes. The stimulator coils must be placed carefully to ensure that session-to-session comparisons are valid. The operational amplifier that powers each vibrating motor is used as a programable current source because it provides a high input signal and changes the current limit according to the conditions. The output current will always be at the specified current limit, which is not a use suggested by the manufacturer. The operational amplifiers generate a large amount of heat; therefore, cooling may become an issue. More efficient methods for powering the vibrating motors, such as a form of pulse width modulation, will be investigated in the future.

## Data Availability Statement

All datasets generated for this study are included in the article/Supplementary Material.

## Ethics Statement

The studies involving human participants were reviewed and approved by the Committee on Human Research Protection at East China Normal University. The patients/participants provided their written informed consent to participate in this study.

## Author Contributions

LW, Y-DZ, HW, XL, and XJ designed the experiments. LW supported the study. LW and XJ wrote the manuscript. XJ and YW performed the experiments and analyzed the data.

## Conflict of Interest

The authors declare that the research was conducted in the absence of any commercial or financial relationships that could be construed as a potential conflict of interest.
