# Supplementary material for: A Simple and Compact MR-Compatible Electromagnetic Vibrotactile Stimulator
Source: Front Neurosci. 2020 Jan 17;13:1403. doi: 10.3389/fnins.2019.01403 (PMC6978794; doi:10.3389/fnins.2019.01403)
Supplement: Supplementary file 1 [file Table_1.docx]

**Table S1. The standard of ROI selection.**

| Side | Brain region | Brodmann area | Talairach coordinates(mm) | | |
| --- | --- | --- | --- | --- | --- |
|  |  |  | x | y | z |
| Right | Postcentral Gyrus | 2 | 49 | -25 | 51 |
| Right | Postcentral Gyrus | 43 | 51 | -15 | 17 |
| Left | Postcentral Gyrus | 40 | -54 | -22 | 19 |
