# Supplementary material for: A Simple and Compact MR-Compatible Electromagnetic Vibrotactile Stimulator
Source: Front Neurosci. 2020 Jan 17;13:1403. doi: 10.3389/fnins.2019.01403 (PMC6978794; doi:10.3389/fnins.2019.01403)
Supplement: Supplementary file 2 [file Table_2.docx]

**Table S2. Accuracy of each pair of stimuli in the vibrotactile frequency discrimination task.**

| S-1(Hz) | S-2(Hz) | Accuracy(mean±SD) |
| --- | --- | --- |
| 40 | 60 | 77.78 ± 13.61% |
| 40 | 80 | 97.22 ± 6.81% |
| 40 | 100 | 100.00 ± 0.00% |
| 60 | 80 | 75.00 ± 13.94% |
| 60 | 100 | 100.00 ± 0.00% |
| 80 | 100 | 61.11 ± 17.21% |
| 60 | 40 | 69.45 ± 12.54% |
| 80 | 40 | 100.00 ± 0.00% |
| 100 | 40 | 100.00 ± 0.00% |
| 80 | 60 | 72.22 ± 20.18% |
| 100 | 60 | 97.22 ± 6.81 % |
| 100 | 80 | 63.89 ± 19.48% |
