# Supplementary material for: A Simple and Compact MR-Compatible Electromagnetic Vibrotactile Stimulator
Source: Front Neurosci. 2020 Jan 17;13:1403. doi: 10.3389/fnins.2019.01403 (PMC6978794; doi:10.3389/fnins.2019.01403)
Supplement: Supplementary file 3 [file Image_1.pdf]

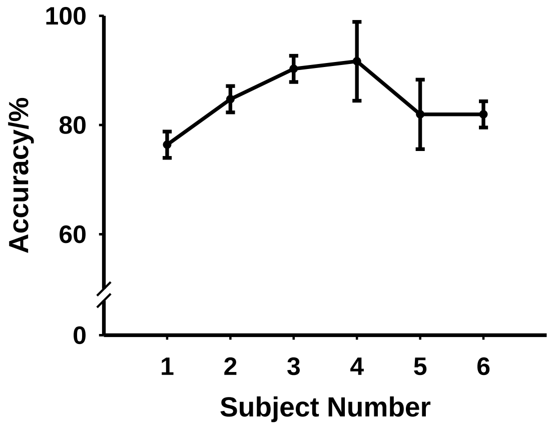

Figure S1. Results from other 6 subjects used in the vibrotactile frequency discrimination task. All subjects were able to discriminate between vibration stimuli. Error bars indicate standard deviation from the mean.
